# Self-Control and Emotion Regulation Mediate the Impact of Karate Training on Satisfaction With Life

**DOI:** 10.3389/fnbeh.2021.802564

**Published:** 2022-01-13

**Authors:** Wiktor Potoczny, Radoslawa Herzog-Krzywoszanska, Lukasz Krzywoszanski

**Affiliations:** Faculty of Pedagogy and Psychology, Institute of Psychology, Pedagogical University of Krakow, Krakow, Poland

**Keywords:** self-control, emotion regulation, self-regulation, satisfaction with life, well-being, karate training, martial arts

## Abstract

Physical activity is an important determinant of a healthy lifestyle. Regular participation in sports-related activities contributes to the maintenance of good psychophysiological and social health. Long-term physical activity has a positive impact on subjective well-being and can reduce stress. Karate is a specific physical activity which focuses on self-regulation and self-development; therefore, it may reduce impulsivity and improve self-control. Good self-control is also related to satisfaction with life and well-being. The presented study aimed to examine the possible intermediate impact of self-control and emotion regulation on the relationship between karate training and satisfaction with life. Fifty-eight karate practitioners and fifty-nine control subjects participated in the research. The Satisfaction With Life Scale and the Brief Self-Control Scale were applied in order to assess life satisfaction and the general level of self-control. The Emotion Regulation Questionnaire was used to assess suppression and reappraisal, both of which are distinct aspects of emotion regulation. The direct and indirect relationships between karate training and satisfaction with life were investigated using a linear regression model that included self-control, suppression and reappraisal as mediating variables. No direct effects of karate training on satisfaction with life were found, whereas karate training was indirectly associated with satisfaction with life via the indirect path that leads through self-control and reappraisal. This indicates that self-control and reappraisal fully mediate the impact of karate training on subjective well-being. Karate training can therefore play an important role in shaping volitional and personality characteristics, both of which contribute to increasing the well-being of trainees.

## Introduction

The issue of life satisfaction has attracted researchers’ attention for several decades. Despite many studies, life satisfaction remains an ambiguous concept and is a current topic of inquiry. Life satisfaction involves evaluating the quality of a person’s life based on this person’s own set of criteria (Pavot and Diener, 1993). Life satisfaction is a key factor in psychological and subjective well-being because it is associated with a positive attitude toward life and the absence of negative feelings (Diener, 1984). The assessment of satisfaction with one’s life may concern various areas of life, such as health, interpersonal relations or financial situation, and it may be formulated in terms of the past, present and future. People combine judgments about various aspects of their lives and integrate them to assess their overall sense of life satisfaction. Generalized satisfaction with life framed in this way can be assessed quantitatively using a single-scale self-report tool (Diener et al., 1999).

Researchers are trying to determine the factors that influence satisfaction with life. Current findings indicate that health status substantially impacts life satisfaction. People who suffer from severe or chronic illnesses have significantly worse well-being and lower life satisfaction (Hutchinson et al., 2004; Strine et al., 2008). Additionally, the lower the life satisfaction, the higher the risk of obesity and health-risk behaviors, such as smoking and alcohol abuse (Strine et al., 2008).

This paper focuses on the possible impact of physical activity and karate training on satisfaction with life. Regular participation in sports training is associated with both physical and psychological components of human health. The results of a meta-analysis conducted by Eime et al. (2013) indicate that physical activity can reduce stress and has a positive impact on satisfaction with life. Other studies also report that long-term physical activity is associated with a higher subjective well-being, and more adaptive responses to negative emotions (Lechner, 2009; Downward and Rasciute, 2011; Asztalos et al., 2012).

Additionally, people who train in sports clubs have a higher level of life satisfaction compared to people who exercise individually (Eime et al., 2010). This result may be explained by the experience of social integration during training, which may enhance well-being (Vilhjalmsson and Thorlindsson, 1992; Eime et al., 2010). Other analyses also indicate that reducing levels of loneliness and fulfilling the need to belong may be significantly related to satisfaction with life (Schumaker et al., 1993; Mellor et al., 2008).

Karate training is a specific physical activity which focuses on the self-regulation and self-development of trainees (Cynarski, 2014). In karate, body training is connected with personality improvement because it focuses on self-awareness and striving to achieve development in each domain of life. Getting rid of aggression, being systematic, and focusing on the goal are principles that students learn on day 1 (Piotrkowicz, 1998). Fuller (1988) noticed that: “*the martial arts may be viewed as formalized, refined systems of human potential training which provide interesting practical models and mechanisms of psychological intervention.*” (pp. 318). This understanding of the role of martial arts indicates the possible importance of karate training for improved psychological well-being.

Both satisfaction with life and karate training are associated with self-control. Self-control can be defined as the ability to change one’s behaviors or reactions and suppress unwanted impulses in order to adapt to a situation. Self-control is particularly relevant to motivational conflicts in which one must resist a pleasurable temptation in order to satisfy a long-term goal (Tangney et al., 2004; Hofmann et al., 2014). Individuals who less frequently control their reactions are considered lower in dispositional self-control (Tangney et al., 2004; Duckworth and Steinberg, 2015). Research conducted by Hofmann et al. (2014) indicates that level of self-control is a significant predictor of well-being and satisfaction with life. Karate training improves trainees’ discipline and self-control (Messaoud, 2015). In their meta-analysis, Vertonghen and Theeboom (2010) analyzed 27 studies on the socio-psychological aspects of martial arts practice among adolescents. Their findings seem to support the hypothesis that training has positive effects on well-being and self-control. The analyzed studies mainly indicate that martial arts practitioners have a higher level of self-regulation and psychological well-being, and a lower level of violence. Other studies also indicate that Eastern martial arts training can directly improve trainees’ self-control (Lakes and Hoyt, 2004). Karate training also effectively reduces the level of aggression and impulsiveness, both of which are negatively related to satisfaction with life (Zivin et al., 2001; MacDonald et al., 2005). This suggests that karate training may indirectly foster the attainment of a high quality of life by having a beneficial effect on self-control.

In addition to self-control, emotion regulation may also play a mediating role in the effects of karate training on life satisfaction. Emotion regulation can be defined as the processes that are responsible for monitoring, evaluating, and modifying emotional responses by initiating, inhibiting, or modulating them (Thompson, 1994; Niven et al., 2009). Gross (1998) concentrated on two common forms of emotion regulation: cognitive reappraisal and expressive suppression. Gross and John (2003) showed that cognitive reappraisal is a more adaptive strategy than suppression since it fosters well-being and interpersonal functioning.

The control of emotional reactions is also important in sports training. Research confirms that people who participate in sports activities have better self-control and use more adaptive forms of emotion regulation (Vertonghen and Theeboom, 2010; Kucharski et al., 2018); moreover, acquiring expertise in sports requires high levels of self-regulation and self-motivation (Kavussanu and Kitsantas, 2011; Kitsantas et al., 2017). Karate, like other physical activities, improves the subjective well-being and general health of participants (Bu et al., 2010; Chang and Jang, 2018), therefore it may have an impact on satisfaction with life. The presented study aimed to examine the possible intermediate impact of self-control and emotion regulation on the relationship between karate training and satisfaction with life. For this purpose, we studied a group of karate practitioners and a control group of non-training subjects. We then attempted to determine whether there are indirect effects in a mediation model in which the explanatory variable is karate training, the mediating variables are self-control and emotion regulation, and the response variable is life satisfaction.

## Method

### Subjects

The minimum sample size needed to detect the effect size of *f*^2^ ≥ 0.15 with probability ≥ 0.9, assuming the type I error rate set at α = 0.05 was 108. The study was attended by 117 people selected on purpose to two groups. 58 subjects (43 males and 15 females; mean age 25.95, with standard deviation 7.37) trained karate in clubs representing different training styles (Oyama and Kyokushin) for at least 1 year (mean 8.07). Another criterion of inclusion was participation in at least one training session per week at the club under the supervision of a coach. The control group consisted of 59 non-training subjects (44 males and 15 females; mean age 26.61, with standard deviation 6.59). These people were mainly students and office workers who did not undertake any regular sports training.

### Measures

#### Satisfaction With Life Scale

The Satisfaction With Life Scale (SWLS), developed by Diener et al. (1985) and adapted into Polish by Jankowski (2015), was used to measure the cognitive-judgmental aspect of subjective well-being. This questionnaire contains five statements (all positively worded) rated on a seven-point Likert-type scale. The summed total score on SWLS ranges from 5 to 35 points: high values reflect high levels of satisfaction with life. The high value (0.86) of Cronbach’s α (Jankowski, 2015) indicates good internal consistency of the Polish version of SWLS. Test-retest reliability in 3-week intervals was 0.85–0.93; in 6-week intervals it was 0.87–0.88; in the 9-week interval, it was 0.86 (Jankowski, 2015).

#### Brief Self Control Scale

Pilarska and Baumaister’s (2018) Polish adaptation of the Brief Self-Control Scale (BSCS) (Tangney et al., 2004) was used to measure dispositional self-control. The BSCS is a thirteen-item self-report questionnaire that consists of four positively worded statements and nine negatively worded statements. The subject is asked to rate each statement on a five-point Likert scale: from 1 (“not at all”) to 5 (“very much”). The BSCS score is computed as the sum of ratings of all items after reverse coding of negatively worded items; high values reflect greater self-control. Pilarska and Baumaister (2018) confirmed that the Polish version of BSCS has good internal consistency (Cronbach alpha:0.84) and satisfactory temporal stability in a 2-week test-retest (Pearson correlation for test-retest:0.87).

#### Emotion Regulation Questionnaire

The Emotion Regulation Questionnaire (ERQ) (Gross and John, 2003), as translated into Polish by Kobylińska (2011) using a back translation procedure, was used to assess two aspects of emotion regulation. This questionnaire consists of ten items that form two scales: “Suppression” (four questionnaire items) and “Reappraisal” (six questionnaire items). The subject responds to each item by providing an answer on a seven-point Likert-type scale (from 1—completely disagree, to 7—completely agree). Higher scores indicate a stronger tendency to use suppression and reappraisal. The internal consistency of the questionnaire, as assessed by Cronbach’s α in a study of 349 people, was 0.77 for Reappraisal and 0.74 for Suppression (Śmieja et al., 2011).

### Statistical Analysis

Means with 95% confidence intervals, medians, standard deviations, and interquartile ranges were calculated to assess the location and dispersion of scores obtained in SWLS, BSCS, and ERQ. Skewness and kurtosis were used to assess the shape and normality of the distribution of scores.

Mediation analysis in a parallel multiple mediation model (MacKinnon, 2012) was conducted to examine whether self-control, suppression and reappraisal mediate the impact of Karate training on satisfaction with life. Parameter estimates for indirect, direct, and total effects were estimated using multiple linear regression fit by ordinary least squares (Hayes, 2018) and were tested for significance using bias-corrected bootstrap confidence intervals (Bollen and Stine, 1990; Shrout and Bolger, 2002; Preacher and Hayes, 2008) with 5,000 replications. Age dichotomized by median split (24 years) and gender were entered into the mediation model as covariates to control their impact on satisfaction with life. Mediation analyses were conducted in jamovi Advanced Mediation Models (jAMM) module (Gallucci, 2021). Jamovi (The jamovi project, 2021) is free and open-source statistical software which is based on the R programming language for statistical computing (R Core Team, 2021).

## Results

Descriptive statistics in the karate training group and the control group for scores in BSCS, ERQ, and SWLS are presented in Table 1.

**TABLE 1:** Descriptive statistics for scores on scales of self-report measures.

Scale	Training	Mean	95% confidence interval for mean		Median	SD	IQR	Skewness		Kurtosis	
			Lower	Upper				Skewness	SE	Kurtosis	SE
BSCS	No	43.59	41.94	45.25	43	6.49	9	–0.256	0.311	–0.279	0.613
	Yes	48.12	46.19	50.05	48	7.49	11.75	–0.126	0.314	–0.686	0.618
ERQS	No	16.48	15.21	17.75	16	4.98	7.5	0.279	0.311	0.111	0.613
	Yes	16.10	14.86	17.38	16	4.97	7.25	0.055	0.314	–0.050	0.618
ERQR	No	28.12	26.60	29.64	28	5.97	7.5	–0.204	0.311	0.261	0.613
	Yes	31.31	30.02	32.60	31.5	4.50	5.75	–0.505	0.314	0.694	0.618
SWLS	No	22.39	21.59	23.19	23	3.15	3	–0.407	0.311	0.685	0.613
	Yes	24.02	23.01	25.02	24	3.91	4	–0.371	0.314	0.660	0.618

*SD, standard deviation; IQR, interquartile range; SE, standard error; BSCS, Brief Self-Control Scale; ERQS, Suppression scale in Emotion Regulation Questionnaire; ERQR, Reappraisal scale in Emotion Regulation Questionnaire; SWLS, Satisfaction with Life Scale.*

More than half of the variance in SWLS scores, *R*^2^ = 0.546, *p* < 0.001, was explained by the full regression model, which was included in the mediation analysis, which estimates the indirect, direct and total effects of karate training on satisfaction with life, controlled for gender and dichotomized age, with self-control, suppression, and reappraisal as mediating variables.

A similar value (*R*^2^ = 0.506, *p* < 0.001) was obtained for the mediation analysis, which was calculated without controlling for gender and age effects; the difference in *R*^2^ between the two analyses was found to be small but statistically significant (Δ*R*^2^ = 0.040, *p* = 0.010). Regression models for the mediating variables were also significant and achieved R-squared values ranging from 0.168 to 0.267 (see Table 2 for details).

**TABLE 2:** Fit measures and overall model tests for linear regression models included in the mediation analysis, which estimates indirect, direct and total effects of karate training on satisfaction with life, with self-control, suppression, and reappraisal as mediating variables, and with gender and dichotomized age as covariates.

					Overall model test			
Model	Dependent variable	R	R^2^	Adjusted R^2^	F	df1	df2	p
Mediator model	BSCS	0.490	0.240	0.220	11.89	3	113	<0.001
	ERQS	0.516	0.267	0.247	13.69	3	113	<0.001
	ERQR	0.410	0.168	0.146	7.619	3	113	<0.001
Full model	SWLS	0.739	0.546	0.521	22.03	6	110	<0.001

**FIGURE 1:**
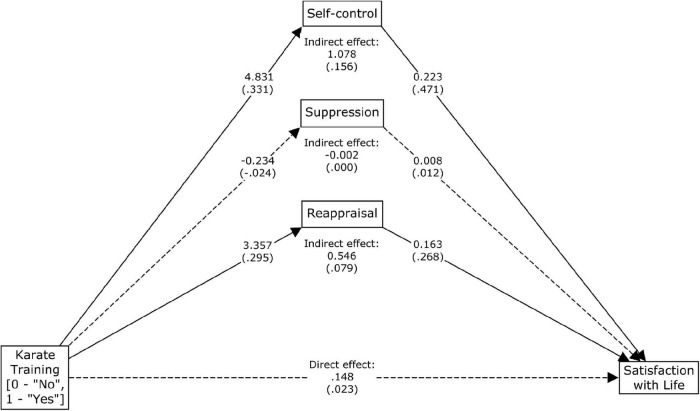
Diagram presenting raw and standardized (in brackets) parameter estimates obtained in the linear regression-based mediation analysis of indirect, direct and total effects of karate training on satisfaction with life with self-control, suppression, and reappraisal as mediating variables, and with gender and dichotomized age as covariates. Solid lines–significant effect; dashed lines–insignificant effect. Parameter estimates were tested for significance using bias-corrected bootstrap confidence intervals. Effects of gender and dichotomized age were estimated in mediation analysis but are not shown.

Table 3 shows the parameter estimates for the indirect, direct, and total effects of karate training on satisfaction with life, controlled for gender and dichotomized age, with self-control, suppression, and reappraisal as mediating variables. The direct effect of karate training on satisfaction with life did not differ significantly from zero. The karate training was indirectly associated with life satisfaction via the indirect paths that lead through self-control and reappraisal. Karate practitioners had higher self-control and reappraisal, which were associated with greater satisfaction with life. The scores on the Suppression scale of ERQ did not mediate the effect of karate training. Females scored significantly higher than males on BSCS, SWLS and the Reappraisal scale of ERQ; they scored significantly lower on the Suppression scale of ERQ. As compared to the younger group, respondents aged 25–45 years scored significantly higher on BSCS and on both scales of ERQ, but not on SWLS (see Table 3 and Figure 1 for details).

**TABLE 3:** Indirect, direct, and total effects of karate training on satisfaction with life in regression-based mediation analysis with self-control, suppression, and reappraisal as mediating variables, and with gender and dichotomized age as covariates.

				95% confidence interval		
Effect type	Effect	Estimate	SE	Lower	Upper	β
Indirect	TRAINING ⇒ BSCS ⇒ SWLS	1.078	0.368	0.505	1.991	0.156
	TRAINING ⇒ ERQS ⇒ SWLS	–0.002	0.048	–0.127	0.081	0.000
	TRAINING ⇒ ERQR ⇒ SWLS	0.546	0.283	0.130	1.254	0.079
Component	TRAINING ⇒ BSCS	4.831	1.188	2.557	7.233	0.331
	BSCS ⇒ SWLS	0.223	0.053	0.118	0.327	0.471
	TRAINING ⇒ ERQS	–0.234	0.789	–1.772	1.289	–0.024
	ERQS ⇒ SWLS	0.008	0.058	–0.106	0.121	0.012
	TRAINING ⇒ ERQR	3.357	0.977	1.386	5.213	0.295
	ERQR ⇒ SWLS	0.163	0.06	0.048	0.286	0.268
Direct	TRAINING ⇒ SWLS	0.148	0.553	–0.980	1.185	0.023
Total	TRAINING ⇒ SWLS	1.770	0.579	0.634	2.905	0.245
Covariates	GENDER ⇒ BSCS	3.203	1.525	0.009	6.048	0.191
	GENDER ⇒ ERQS	–5.082	0.971	–6.974	–3.193	–0.450
	GENDER ⇒ ERQR	2.591	0.915	0.803	4.423	0.199
	GENDER ⇒ SWLS	1.717	0.592	0.598	2.936	0.217
	AGE_D ⇒ BSCS	5.249	1.281	2.761	7.816	0.358
	AGE_D ⇒ ERQS	1.904	0.832	0.256	3.541	0.192
	AGE_D ⇒ ERQR	2.925	1.004	0.957	4.887	0.256
	AGE_D ⇒ SWLS	0.893	0.541	–0.154	1.968	0.129

*SE, standard error; β, standardized parameter estimate; BSCS, Brief Self-Control Scale; ERQS, Suppression scale in Emotion Regulation Questionnaire; ERQR, Reappraisal scale in Emotion Regulation Questionnaire; SWLS, Satisfaction with Life Scale. Coding for TRAINING: 0 | 1 (No | Yes). Coding for GENDER: 0 | 1 (Male | Female). AGE_D: Age in years dichotomized by a median split, coded 1 | 2 (18–24 years, n = 63 | 25–45 years, n = 54).*

## Discussion

The current study is the first to explore the intermediate impact of self-control and emotion regulation on the relationship between karate training and satisfaction with life. In our research, we tested whether self-control and emotion regulation help to understand the relationship between sport training and satisfaction with life.

We found that karate training is indirectly related to satisfaction with life through the indirect pathways that lead through self-control and cognitive reappraisal. That is, karate training is associated with high self-control and high cognitive reappraisal, which consequently increase life satisfaction. Our results are consistent with those obtained in previous studies, which showed that karate training increases quality of life and psychological well-being (Marie-Ludivine et al., 2010; Jansen et al., 2017) and enhances self-control (Lakes and Hoyt, 2004; Vertonghen and Theeboom, 2010; Messaoud, 2015). Sport practitioners use a wide range of flexible and effective emotion regulation strategies in response to the changing contexts and demands of situations (Kucharski et al., 2018). Additionally, it has been suggested (Madden, 1995) that the psychological benefits of martial arts training are greater than the benefits of other forms of physical activity. Coupled with our results, the association between self-control and well-being (Hofmann et al., 2014) suggests that karate training substantially affects life satisfaction.

Our results show that cognitive reappraisal mediated the effect of karate training on satisfaction with life, as opposed to expressive suppression, which did not mediate this effect. Previous findings also showed that cognitive regulatory strategies, such as reappraisal, can improve mood and foster emotional well-being (Balzarotti et al., 2016). Studies by Gross and John (2003) indicated that people who prefer reappraisal can cope with negative emotions more effectively and maintain better quality of interpersonal relationships. In contrast, the use of expressive suppression is associated with reduced well-being (Haga et al., 2007). The assessment of only the positive dimension of psychological well-being could explain that expressive suppression does not mediate the relationship between karate training and satisfaction with life. Meanwhile, expressive suppression, which is a non-adaptive form of emotion regulation could mediate the relationship between karate training and the negative aspect of psychological well-being (i.e., reduced quality of life). However, this supposition also requires further research, using tools to assess the negative aspects of psychological well-being, e.g., depressive symptoms or distress, etc. Coupled with our results, this suggests that only a more adaptive form of emotion regulation provides an intermediate link between karate training and high life satisfaction.

Our results indicate that karate training can have a positive impact on satisfaction with life. It was also shown that eastern martial arts training helps to significantly improve participants’ self-control and reduce levels of aggression and impulsivity (Zivin et al., 2001; Vertonghen and Theeboom, 2010). Furthermore, a study conducted by Kimberley D. Lakes and William T. Hoyt (Lakes and Hoyt, 2004) indicates that adolescents participating in a 3-month intervention using martial arts training (Tae Kwon Do) scored better in self-control than those participating in standard physical activity training. Martial arts training may have beneficial effects on participants’ self-control in various contexts. Therefore, karate training should be more widely included in extracurricular activities for children and adolescents. Karate training is especially useful for people prone to aggressive and impulsive behavior.

### Strengths, Limitations, and Further Research Directions

This study examined relationships that had not been previously analyzed together and found that self-control and reappraisal fully mediate the impact of karate training on satisfaction with life. More than half of the variance in the dependent variable was accounted for by our mediation model, thus indicating that self-control and reappraisal explain substantially higher levels of trainees’ satisfaction with life. We showed that the association between karate training and subjective well-being and life satisfaction can be explained by self-control and cognitive reappraisal.

Our study had also some limitations. First, since our study was preliminary and exploratory, it was conducted on a small sample which was taken from a small geographic area. A gender disparity in our sample was also observed. However, the gender proportions in our sample are consistent with the number of women in the Polish population of karate practitioners. A report by the Polish Central Statistical Office (Malinowska, 2019) shows that women make up approximately 30% of total karate trainees in Poland. In further studies, the sample size should be larger, and more women should be surveyed to examine whether gender moderates the relationships between karate training and satisfaction with life that are mediated by self-control and emotional regulation. Secondly, only a small range of controlled sociodemographic variables was included in our study; therefore, variables such as education, household situation, and material and professional status should also be included in future research. Moreover, comparing martial arts practitioners to other athletes should also be considered in future studies.

## Data Availability Statement

The raw data supporting the conclusions of this article will be made available by the authors, without undue reservation.

## Ethics Statement

Ethical review and approval was not required for the study on human participants in accordance with the local legislation and institutional requirements. The patients/participants provided their written informed consent to participate in this study.

## Author Contributions

WP, LK, and RH-K developed the study concept and drafted the first manuscript. WP organized and conducted the survey. RH-K supervised the research. LK performed the statistical analysis, prepared the figure, and obtained the funding for publication costs. All authors contributed equally to the revision of the manuscript and approved the submitted version.

## Conflict of Interest

The authors declare that the research was conducted in the absence of any commercial or financial relationships that could be construed as a potential conflict of interest.

## Publisher’s Note

All claims expressed in this article are solely those of the authors and do not necessarily represent those of their affiliated organizations, or those of the publisher, the editors and the reviewers. Any product that may be evaluated in this article, or claim that may be made by its manufacturer, is not guaranteed or endorsed by the publisher.
